# Food Cravings and Obesity in Women with Polycystic Ovary Syndrome: Pathophysiological and Therapeutic Considerations

**DOI:** 10.3390/nu16071049

**Published:** 2024-04-03

**Authors:** Katerina Stefanaki, Dimitrios S. Karagiannakis, Melpomeni Peppa, Andromachi Vryonidou, Sophia Kalantaridou, Dimitrios G. Goulis, Theodora Psaltopoulou, Stavroula A. Paschou

**Affiliations:** 1 Endocrine Unit and Diabetes Center, Department of Clinical Therapeutics, Alexandra Hospital, School of Medicine, National and Kapodistrian University of Athens, 11527 Athens, Greece; k.stefanaki@hotmail.gr (K.S.); tpsaltop@hotmail.com (T.P.); s.a.paschou@gmail.com (S.A.P.); 2 Academic Department of Gastroenterology, Laiko General Hospital, School of Medicine, National and Kapodistrian University of Athens, 11527 Athens, Greece; 3 Endocrine Unit and Diabetes Center, Second Department of Internal Medicine, Attikon University Hospital, School of Medicine, National and Kapodistrian University of Athens, 11527 Athens, Greece; moly6592@yahoo.com; 4 3rd Department of Internal Medicine, Sotiria Chest Disease Hospital, Medical School, National and Kapodistrian University of Athens, 11527 Athens, Greece; 5 Department of Endocrinology and Diabetes Center, Hellenic Red Cross Hospital, 11526 Athens, Greece; mahi_vr@hotmail.com; 6 3rd Department of Obstetrics and Gynecology, Attikon University Hospital, School of Medicine, National and Kapodistrian University of Athens, 11527 Athens, Greece; sophiakalantaridou@gmail.com; 7 Unit of Reproductive Endocrinology, First Department of Obstetrics and Gynecology, School of Medicine, Aristotle University of Thessaloniki, 57001 Thessaloniki, Greece

**Keywords:** polycystic ovary syndrome, obesity, food disorders, binge eating, food cravings

## Abstract

Polycystic ovary syndrome (PCOS), the most common endocrine disorder in women of reproductive age, constitutes a metabolic disorder frequently associated with obesity and insulin resistance (IR). Furthermore, women with PCOS often suffer from excessive anxiety and depression, elicited by low self-esteem due to obesity, acne, and hirsutism. These mood disorders are commonly associated with food cravings and binge eating. Hypothalamic signaling regulates appetite and satiety, deteriorating excessive food consumption. However, the hypothalamic function is incapable of compensating for surplus food in women with PCOS, leading to the aggravation of obesity and a vicious circle. Hyperandrogenism, IR, the reduced secretion of cholecystokinin postprandially, and leptin resistance defined by leptin receptors’ knockout in the hypothalamus have been implicated in the pathogenesis of hypothalamic dysfunction and appetite dysregulation. Diet modifications, exercise, and psychological and medical interventions have been applied to alleviate food disorders, interrupting the vicious circle. Cognitive–behavioral intervention seems to be the mainstay of treatment, while the role of medical agents, such as GLP-1 analogs and naltrexone/bupropion, has emerged.

## 1. Introduction

Polycystic ovary syndrome (PCOS) is the most common endocrine disorder in women of reproductive age, with a worldwide prevalence of between 9 and 18% [1,2,3]. According to the 2018 International Guidelines, PCOS may be diagnosed if any two of the following are present: (a) clinical or biochemical hyperandrogenism, (b) oligo-anovulation (menstrual cycle duration >35 days or <8 menstrual episodes per year), (c) polycystic-appearing ovarian morphology on an ultrasound, with the exclusion of other relevant disorders [4,5]. Apart from a reproductive disorder, PCOS has been considered a metabolic disease, strongly associated with insulin resistance (IR), metabolic syndrome (MS), and type 2 diabetes mellitus (T2DM). Indeed, individuals with PCOS have a 5.46-fold higher risk of developing IR compared to the general population and a 4.35- and 23.46-fold higher risk of T2DM and MS, respectively [6]. Obesity is believed to play a central role in the development of IR in PCOS, as many women with this condition are reported to be overweight or obese [7]. However, the existence of IR in lean PCOS also indicates that other factors are involved [6]. Interestingly, defective post-receptor signaling and the decreased insulin receptor beta subunit in skeletal muscle, liver, adipose tissue, and kidneys have been implicated in the pathogenesis of IR [8].

On top of hyperandrogenemia and metabolic derangements, PCOS is associated with several psychological disorders, such as anxiety, mood instability, and depression [9]. Sexual and relational dysfunction, femininity and fertility concerns, loss of self-confidence, and negative body image due to obesity, acne, and hirsutism are potential causative factors. Hyperinsulinemia, high testosterone concentrations, and the impaired secretion of ghrelin and cholecystokinin (CKK) contribute to the pathophysiology of PCOS [10]. Subsequently, psychological and mood disturbances predispose to binge eating and food cravings, which, in turn, further aggravate obesity and IR, resulting in a relentless vicious circle [11].

Although the association between PCOS and food cravings has been verified, the exact pathogenetic mechanism has yet to be elucidated. Hence, the scope of this review is to display recent data regarding the metabolic and hormonal disorders that predispose to the development of food disorders in PCOS. Additionally, it addresses nutritional and lifestyle modifications to alleviate food disorders and improve the quality of life in women with PCOS.

## 2. Metabolic and Hormonal Derangements Predisposing to Food Disorders in Women with PCOS

### 2.1. Obesity

Obesity is a significant risk factor for PCOS, with up to 80% of females with PCOS experiencing obesity [7], though lean women can still be diagnosed with the disease [12]. Interestingly, reproductive issues are more frequently discovered in obese females, regardless of PCOS. Compared to women with a healthy weight, obese women are more likely to experience menstrual irregularities and infertility due to lack of ovulation [13]. To investigate the relationship between obesity and PCOS, studies have focused on girls and adolescents to understand which condition comes first. Researchers have observed that girls who had a high body mass index (BMI) during childhood are more likely to develop oligomenorrhea and be diagnosed with PCOS in young adulthood. However, it is unclear whether these girls already had signs of PCOS during childhood. A study was conducted to determine whether PCOS in adolescents can predict obesity. PCOS was diagnosed using the Rotterdam criteria in 12 out of 30 (40%) oligomenorrheic girls at the age of 14 years. By age 24, 33% of the girls with PCOS displayed class III obesity, compared to 8.4% of those without PCOS [14]. On the other hand, a study was conducted on 244 randomly selected girls who had reached puberty to investigate the effect of obesity on the development of abnormal ovarian morphology. The results showed that 61.1% of the obese girls developed PCOS compared to 2.1% of the normal-weight subjects [15]. These studies show that there is a correlation between obesity and PCOS in their development. However, it is not fully understood how obesity explicitly affects the pathophysiology of PCOS. Obesity is linked to IR and higher insulin levels, which can lead to the increased production of ovarian androgens [16]. Excess adipose tissue leads to androgens’ aromatization to estrogen, negatively affecting the hypothalamic–pituitary–ovarian (HPO) axis and gonadotropin production [17].

### 2.2. Insulin Resistance (IR)

As a response to dietary stimuli, insulin is secreted by the beta pancreatic cells to maintain glucose homeostasis [18]. Insulin promotes glucose uptake via the muscle cells, drives protein synthesis, inhibits lipolysis, increases fatty acid and glucose uptake in adipose tissue, and suppresses glucose production in the liver [19]. After binding on surface receptor tyrosine kinase, insulin activates AKT serine/threonine kinase 2 (AKT2). This binding promotes the translocation of GLUT4-containing storage vesicles (GSVs) to the plasma membrane, permitting the entry of glucose into the cells. The activation of AKT2 also enhances glycogen synthesis via glycogen synthetase (GS) [20,21,22]. However, insulin signaling may be blunted under certain circumstances, leading to IR. In such a case, the diacylglycerol (DAG)-mediated activation of protein kinase C theta (PKCθ) is demonstrated, leading to the impairment of insulin signaling. In contrast, an increased sequestration of AKT2 by protein kinase C zeta (PKCζ) also occurs. Impaired AKT2 activation reduces GSVs’ migration to the plasma membrane, impairing glucose uptake. Moreover, AKT2 dysfunction negatively affects insulin-mediated glycogen synthesis [20,22]. Consequently, β-cells overreact and produce more insulin to overcome and compensate for IR. Hence, hyperinsulinemia is the hallmark of IR, at least at the initial stages of the disease.

Free fatty acids (FFAs) and their metabolites, which are elevated in obesity due to excessive lipolysis of the subcutaneous and visceral adipose tissue, disrupt the intracellular action of AKT2 [23,24]. Additionally, the dysregulation of adipokines, such as adiponectin, has been implicated. Typically, adiponectin improves insulin sensitivity by activating AMP-activated protein kinase (AMPK) and increasing fat oxidation [25]. However, adiponectin concentrations are lower in women with PCOS than in the controls, worsening IR [26].

“Hepatokine” proteins secreted by the liver, play a crucial role in glucose and lipid metabolism, contributing to the pathogenesis of IR [27]. Interestingly, some hepatokines aggravate, and others attenuate the degree of IR. Fibroblast growth factor 21 (FGF-21), sex hormone-binding globulin (SHBG), and angiopoietin-like proteins (ANGPTLs) belong to hepatokines with a positive metabolic impact. At the same time, fetuin-A and B, lipocalin 2 and 13 (LCN2, LCN13), hepassocin, and selenoprotein P (SEPP1) comprise a group of hepatokines with adverse effects [28]. According to a recent study by Giannouli et al., PCOS patients have lower levels of selenoprotein P and SHBG in their blood compared to the controls. This study also found a positive correlation between selenoprotein P and testosterone levels (r = 0.325, *p* = 0.007) and the free androgen index (r = 0.361, *p* = 0.002). Furthermore, FGF21 concentrations were shown to be associated with an increased risk of fatty liver disease in PCOS subjects [29]. A recent cross-sectional study compared 45 women with PCOS to 42 healthy controls aged between 18 and 45. The study found higher levels of *C*-reactive protein (CRP) and hepassocin in the group with PCOS. Additionally, the study revealed a significant positive correlation between the hepassocin and luteinizing hormone (LH) and a significant negative correlation between hepassocin and BMI, waist circumference (WC), fat ratio, and glycated hemoglobin (HbA1c) [30].

IR has been associated with increased serum concentrations of inflammatory markers, such as CRP [31]. Notably, the association between inflammation-circulated pro-inflammatory cytokines and IR is bidirectional. Research in rats has shown that IR precedes the production of pro-inflammatory cytokines [32], but the latter predisposes to the further aggravation of IR. Cytokines may inhibit the AKT signaling pathway, either directly or indirectly, by promoting lipolysis, resulting in increased FFA concentrations. FFP may negatively affect the intracellular action of phosphoinositide 3-kinases (PI3K) and AKT [23,24].

IR and concurrent hyperinsulinemia stimulate the pituitary production of LH, resulting in the increased production of ovarian androgens [16]. Furthermore, insulin can directly stimulate ovarian theca cells to produce and release androgens and exacerbate adrenal androgen production [33,34]. Interestingly, there appears to be a bidirectional connection between IR and hyperandrogenemia. Overexposure to androgen has been linked to the malfunctioning of islets of Langerhans, resulting in compromised pancreatic metabolic functions and causing hyperinsulinemia [34]. A study of adolescent girls with PCOS found that free testosterone was an independent risk factor for developing MS and IR [35]. Similarly, women with PCOS and MS had significantly higher free testosterone levels than women with PCOS but without metabolic syndrome after adjusting for BMI [36]. The link between androgens and IR has been established in women undergoing gender reassignment. They developed IR after four months of receiving the intramuscular administration of testosterone esters [37]. Similarly, oral methyltestosterone was found to induce IR in healthy premenopausal women, as assessed by a clamp after 10–12 days [38]. The hypothalamus, particularly the arcuate nuclei (ARC), is an area of the central nervous system (CNS) that regulates appetite, food consumption, and energy homeostasis [39]. The hypothalamic neurons respond to peripheral nutrients, such as glucose and fatty acids, and hormones, such as insulin, leptin, ghrelin, adiponectin, resistin, and ovarian steroids [40,41,42,43,44].

Insulin influences the hypothalamic function by connecting to insulin-specific neural receptors and modulating eating behavior and body weight [45]. To be precise, insulin activates the hypothalamus, and the consecutive efferent autonomic nervous signaling towards peripheral tissues controls satiety, hepatic glucose production, pancreatic insulin secretion, and general insulin sensitivity [46]. Indeed, a blunted brain response to insulin, called “brain IR” is associated with the dysregulation of food intake and glucose metabolism, fostering overeating, weight gain, and the aggravation of peripheral IR.

The mechanisms implicated in the pathogenesis of peripheral IR participate in the pathogenesis of brain IR. Hence, obesity disturbs the production of LH, as reduced pulsatile amplitude of LH has been identified in animal models fed with a high-fat diet and in obese women, indicating a hypothalamic–pituitary dysfunction [47,48].

Moreover, systemic inflammation due to IR generates a local inflammatory response in the hypothalamus, as demonstrated by an increased number of macrophages in the hypothalamus of male mice [49,50]. In addition, hyperinsulinemia provokes brain insulin receptor downregulation, resulting in the lower sensitivity of the hypothalamus to insulin signals [51,52].

### 2.3. Gastrointestinal Hormones

Apart from brain IR, the effectiveness of leptin, a protein that inhibits food intake and increases energy expenditure, is blunted in PCOS [53]. Leptin is associated with body weight, BMI, and IR parameters such as HOMA-IR and QUICKI [54]. It is elevated in women with PCOS, whether they are obese or lean, as a compensatory mechanism for obesity or IR (the latter may implicate PCOS even in the absence of obesity) [55]. However, despite its elevation, leptin cannot prevent excessive food intake [56]. This fact has been attributed to leptin resistance caused by reduced leptin receptors in the hypothalamus [57].

Ghrelin is another hormone that regulates appetite and food intake. It is particularly increased pre-prandially, producing an orexigenic effect, while it decreases postprandially to cease food consumption [58]. Although an argument exists regarding whether ghrelin concentrations are lower in women with PCOS compared to healthy controls [59,60], a lower reduction in postprandial ghrelin concentrations has been identified in women with PCOS compared to weight-matched healthy controls [61,62,63]. Moreover, Saydam et al. found that women with PCOS had either decreased or unaltered fasting ghrelin concentrations and either a decreased or unaltered postprandial suppression of CCK compared to healthy controls [64]. This abnormality increases the risk of excessive food intake.

Furthermore, animal studies have shown that estradiol (E_2_) and CCK act synergically on the nucleus of the solitary tract (NTS) to induce satiety [65,66]. In subjects with PCOS, reduced postprandial CCK secretion is associated with increased concentrations of testosterone [67]. Aside from the synergistic action between E_2_ and CCK, the former promotes an anorexigenic effect in animal models and controls binge eating by stimulating the serotonin (5-HT) neurons in the dorsal raphe nuclei (DRN) [68].

### 2.4. Brain Reward System

The mesolimbic system, also known as the reward system, comprises the striatum, prefrontal cortex, amygdala, and hippocampus. It plays a role in the physiological and cognitive processing of rewards, a natural process of brain-associated stimuli, such as substances, events, or activities, with a positive or desirable outcome. This phenomenon causes a person to modify their behavior to obtain a positive stimulus. The reward mechanism involves the synchronized discharge of different neurotransmitters, such as 5-HT and endocannabinoids, but dopamine holds a key position in the reward value of food [69,70]. Research has shown that increased cortisol secretion from the upregulation of the hypothalamic–pituitary–adrenal (HPA) axis in acute and chronic stress inhibits dopamine release in the reward system [71]. On the other hand, E2 seems to promote dopamine signaling [72]. In PCOS patients, increased androgens downregulate the reward system via HPA activation [73]. In addition, brain IR has been linked to alterations in dopamine turnover, causing behavioral and food disorders [74,75].

### 2.5. Hyperandrogenism

Hyperandrogenism constitutes a major pathogenetic component of PCOS and is directly associated with IR and hyperinsulinemia. Insulin binds to insulin receptors on ovarian theca cells and promotes androstenedione production. Besides ovarian production, hyperinsulinemia stimulates androgen synthesis in the adrenal glands [33,34]. Furthermore, insulin interacts with LH and human chorionic gonadotropin (hCG), increasing CYP17A1 activity and, thus, circulating CYP17 concentrations, leading to increased androgen production [76,77]. In addition, insulin inhibits the hepatic production of sex hormone-binding globulin (SHBG), resulting in higher concentrations of free and bio-available testosterone [78]. Androgens induce abdominal adipose accumulation [79], aggravating IR [80,81]. Corbould et al. [81] found that testosterone can inhibit insulin-stimulated glucose uptake by impairing the phosphorylation of PKCζ in women with obesity.

## 3. Eating Disorders in PCOS: Pathogenetic Mechanism

Aside from endocrine and metabolic disease, PCOS is often associated with psychological disturbances and food disorders. Excessive anxiety, depression, mood alterations, anorexia nervosa (AN), bulimia nervosa (BN), food cravings, binge eating, night-eating syndrome, and emotional eating are commonly presented [82,83].

The underlying mechanisms of eating disorders (ED) in PCOS are complex and multifactorial. Excessive stress and negative emotions about body image because of obesity, acne, hirsutism, and menstrual irregularities predispose the development of ED in subjects with PCOS [10]. Furthermore, hyperandrogenism exacerbates ED since testosterone increases anxiety and depression and stimulates appetite and bulimic behavior [9,73,84]. The study by Morgan et al., which investigated the role of facial hirsutism (a standard component of hyperandrogenism) in the development of ED, concluded that the prevalence of ED was 36.3% in women with hirsutism, accompanied by depression, anxiety, low self-esteem, and poor social adjustment [85].

Besides psychological factors and hyperandrogenism, the impaired hypothalamic function plays an essential role in the pathogenesis of appetite dysregulation. To be precise, a two-hit hypothesis has been speculated. Initially, IR and consequent hyperinsulinemia decrease postprandial glucose concentrations, leading to binge eating, the increased consumption of carbohydrates, and weight gain [86]. Subsequently, obesity and hyperandrogenism trigger anxiety disorders and depression, aggravating further ED [87]. However, blunted hypothalamic function due to brain IR, leptin resistance, and the reduced postprandial production of CCK cannot regulate hunger and satiety, resulting in a vicious circle of bulimia, increased food intake, obesity, IR, and mood disorders.

## 4. Eating Disorders: Prevalence and Clinical Aspects

### 4.1. Binge Eating

Binge eating disorder (BED) is one of the most prevalent EDs in PCOS women [88]. According to the fifth edition of the Diagnostic and Statistical Manual of Mental Disorders (DSM), BED is an avoidant–restrictive food intake disorder characterized by large amounts of food consumption in a short period, with a loss of control at least once per week for three months [83]. Recent studies have shown that 33% of women with PCOS have abnormal eating patterns, and 6% have scores suggestive of clinical BED [89]. In addition, Wylie et al. reported that PCOS women present an increased frequency of eating and consume significantly more sweet snacks, with a higher body mass index (BMI) positively associated with eating frequency [90]. In contrast, De Giuseppe et al. reported a higher tendency of under-reporting the consumption of carbohydrates [91]. Another study showed that the prevalence of clinical and subclinical bulimia nervosa is increased among women with PCOS compared to healthy women [92]. A cross-sectional study confirmed a higher prevalence of BED, depression, and overall impaired quality of life in women with PCOS [93]. Following these findings, researchers conducted an online survey concerning BED and food cravings (FCs). They concluded that obesity has a predominant role in EDs in PCOS since 60% of women with obesity and PCOS developed BED and higher mean food craving trait scores [11]. These findings were confirmed by a recent study that pointed to obesity and hyperandrogenism as risk factors for depression and food cravings in women with PCOS [9]. Nevertheless, not all of these studies agree about the possible correlation between obesity and ED. Lee et al. clarified a higher risk of women with PCOS developing EDs, independent of the presence of obesity. The investigators favored body image and low self-esteem as causative factors for EDs [94]. Most studies have documented that BED is more common in PCOS than in healthy individuals. The discrepancy in its prevalence has been attributed to different tests that evaluate EDs [86].

### 4.2. Mood Disorders—Anxiety—Depression

There is a well-established correlation between mood disorders, anxiety, depression, and EDs [95]. Concerning women with PCOS, a cohort study by Hollinrake et al. showed a 4.23-fold higher risk of depressive symptoms independent of obesity and infertility and a 5.11-fold higher risk of developing depression [96]. Likewise, Annagor et al. verified that untreated PCOS women confer an additional risk for psychiatric disorders, such as depression, anxiety, and BED [97]. Furthermore, evidence from a nationwide Swedish cohort revealed an increased risk [odds ratio (OR) 1.56, 95% confidence interval (CI) 1.51–1.61] of developing at least one psychiatric disorder among bulimia, schizophrenia, bipolar disorder, depression, anxiety disorders, and personality disorders in women with PCOS [98]. A meta-analysis of six studies, including 661 women (343 with PCOS and 318 controls), evaluated psychological disorders in PCOS and identified a higher risk of anxiety and depression compared with the controls (OR 2.76; 95% CI 1.26–6.02 and OR 3.51; 95% CI 1.97–6.24, respectively); advanced age, obesity, and clinical hyperandrogenism were the predisposing factors [99]. Adding to the above findings, a recent meta-analysis showed an increased pool prevalence of depression (42%, 95% CI 33–52%), considering the results of 24 cross-sectional studies, and an increased pool prevalence of anxiety (37%, 95% CI 14–60%) based on the results of 16 cross-sectional studies [100].

## 5. Nutritional and Lifestyle Modifications to Improve Food Cravings

Protein-enriched diets (PEDs) benefit glucose homeostasis in patients with T2DM [101,102,103]. Specifically, protein ingestion correlates with an increased intestinal release of CCK and glucagon-like peptide-1 (GLP-1), contributing to reduced food intake via their action on the hypothalamus [104]. A recent meta-analysis demonstrated that higher-protein diets of 1.2–1.6 gr protein/kg/day, with protein quantities of at least 25–30 gr per meal, provide a decrease in food intake and better body weight management compared to non-protein-rich diets [103].

Colombarolli et al. investigated the role of a low-carbohydrate diet in food cravings. They found that a carbohydrate-restricted diet and intermittent fasting are related to increased rates of binge eating and food cravings compared to non-dieters [105]. However, these results were not confirmed in a recent randomized controlled trial (RCT) conducted in overweight or obese patients with T2DM, where no difference was reported between a low-fat, high-protein diet and a low-fat, high-carbohydrate isocaloric diet for a reduction in sweet cravings, fast-food cravings, and the Food Cravings Questionnaire-Trait (FCQ-T), for a similar loss of weight [106].

An enriched-in-soluble fiber diet can also prevent excessive caloric intake. Gastric distention delayed gastric emptying, with increased amounts of unabsorbed nutrients reaching the ileum, and the stimulation of CCK, GLP-1, and peptide YY secretion are among the mechanisms that decrease hunger and prolong satiety. A recent meta-analysis of 15 RCTs verified a significant effect of viscous fiber on body weight, body fat index, and BMI but not on weight circumference [107]. Furthermore, the EPIC-InterAct, a multicenter study of 26,088 participants, showed reduced T2DM risk with total fiber consumption, vegetable fiber, and cereal fiber consumption when adjusted for dietary and lifestyle factors. Between a high and a low-fiber diet, the relative risk of T2DM was lower by 18% in the former (*p* = 0.02) [108]. Likewise, the meta-analysis of Li et al. identified an association between wholegrain intake and lower fasting glucose, lower HbA_1c_, and higher insulin sensitivity [109].

Physical activity is an effective way to manage stress, which significantly contributes to food cravings. However, there are no studies specifically investigating the role of exercise and its type in eliminating food cravings and binge eating in PCOS. The 2018 international evidence-based guidelines recommend undertaking at least 150 min of moderate or 75 min of vigorous exercise per week to prevent weight gain. For weight loss and to prevent weight regain, they recommend undertaking at least 250 min of moderate or 150 min of vigorous exercise per week [110]. Aerobic exercise has been shown to improve IR and insulin sensitivity, as measured by the HOMA-IR and insulin sensitivity index, respectively. Additionally, it can help decrease WC and BMI [111,112,113]. High-intensity interval training (HIIT) alone may improve IR and BMI [114]. In contrast, resistance training alone is ineffective at reducing body weight and BMI [112,113]. Notably, RCTs on the role of exercise in mitigating food cravings in PCOS women are missing.

Behavioral treatments play a central role in the alleviation of food disorders and binge eating. In a meta-analysis of 81 RCTs and 7515 patients, cognitive-behavioral therapy (CBT) was associated with reduced binge-eating episodes and more prolonged abstinence from binge eating, followed by structured self-help treatment. On the contrary, pharmacotherapy and pharmacological weight loss treatment had negligible effects. However, the quality of evidence regarding binge eating outcomes was deficient, whereas the heterogeneity among the included studies was high [115]. The positive impact of CBT on binge eating has been supported by the results of the meta-analysis by Ghaderi et al., where patients treated with CBT presented fewer episodes of binge eating, an improvement in depression, and an improvement in the Eating Disorder Examination Questionnaire (EDE-Q) compared with untreated subjects [116]. Recently, an RCT compared the effect of physical exercise plus dietary therapy (PED-t) to CBT in the treatment of binge eating disorder. According to the results, PED-treated patients showed a faster improvement in EDE-Q and the Clinical Impairment Assessment (CIA) than CBT-treated patients, but the difference was not maintained during the follow-up. Regarding the Beck Depression Inventory (BDI) questionnaire, a post-treatment improvement compared to the baseline was mentioned only in the PED-t arm [117].

## 6. Medical Interventions

The GLP-1 analogs liraglutide and semaglutide at doses of 3 mg/day and 2.4 mg/week, respectively, have been approved by the FDA for the treatment of obesity after the results from animal and human studies showing an anorectic effect and positive metabolic control activity. These agents regulate appetite and food desirability by acting on the hypothalamus, where GLP-1 receptors are expressed [118]. In women with overweight/obesity and PCOS, liraglutide promotes higher weight loss rates than the combination of orlistat plus metformin, according to a meta-analysis of 23 studies and 941 participants. Regarding food cravings, Tronieri JS et al. showed in an RCT that the combination of liraglutide and behavior treatment was superior to behavior treatment alone in reducing hunger and food preoccupation (−16.8 ± 4.0 vs. −0.3 ± 4.2 mm; *p* = 0.005 and −16.3 ± 3.6 vs. 0.2 ± 3.7 mm; *p* = 0.002, respectively), and increasing a sense of fullness (9.8 ± 3.0 vs. −5.1 ± 3.2 mm; *p* = 0.001) at week 6. Notably, the difference remained through week 24 but diminished through week 52 [119]. Likewise, according to the Control of Eating Questionnaire (CoEQ), semaglutide was associated with lower hunger, better control of eating, and fewer food cravings compared to a placebo (*p* < 0.05 for all comparisons) when administrated in patients with obesity for 20 weeks [120]. Moreover, Richards et al. retrospectively found that semaglutide improved binge eating scores compared with topiramate (a drug that reduces appetite). In contrast, the administration of topiramate in semaglutide-treated patients did not offer any additional benefit [121].

Bupropion/naltrexone, a combination treatment proven effective for weight loss, improved aggressive eating behavior, such as binge or emotional eating, cravings for carbohydrates, and post-dinner eating in a clinical trial comparing 23 patients with binge eating disorders versus a control group of 20 individuals with without eating disorders. The positive effect of this treatment occurs by interacting with the hypothalamus-regulated reward circuit [122]. Promising results from the use of bupropion/naltrexone were verified by RCTs, which compared this combination to behavioral intervention in patients with binge eating disorders. In the study by Grilo et al., naltrexone/bupropion was superior to the placebo in alleviating binge eating (OR 2.19, 95% CI 1.03–4.63, *p* = 0.04) but inferior to behavior intervention (remission rates: 17.7% in placebo, 31.3% in naltrexone/bupropion, 37.1% behavior treatment, 57.1% in the combination of naltrexone/bupropion plus behavior treatment; OR 2.84, 95% CI 1.34–6.03, *p* = 0.006 for treatments including behavior intervention compared with treatments not including behavior intervention). Higher remission rates using naltrexone/bupropion compared with the placebo were not preserved beyond the first two months of treatment [123]. Subsequently, in a randomized, double-blind, placebo-controlled, 12-week trial testing naltrexone/bupropion for binge eating, the same investigators showed no differences between naltrexone/bupropion therapy and the placebo regarding binge eating remission [124]. Furthermore, the inability of naltrexone/bupropion to maintain binge eating abstinence was verified in a recent RCT, which compared naltrexone/bupropion versus placebo as binge eating maintenance treatment in patients previously responded to acute treatment with naltrexone/bupropion and behavioral therapy. Binge eating remission rates following 16-week maintenance treatment did not differ between the placebo and the naltrexone/bupropion arm (50.0% vs. 68.8%, respectively; *p* = 0.14). However, the degree of remission maintenance in the naltrexone/bupropion group was correlated to the type of treatment initially given to achieve binge eating remission (i.e., patients who had received naltrexone/bupropion as an initial treatment and the placebo afterward had a lower probability of maintaining the remission, whereas the probability was higher in those being treated with naltrexone/bupropion during both phases) [125].

Several herbal remedies have been used to alleviate IR and infertility in women diagnosed with PCOS [126]. Recent studies have found that including herbal formulas in infertility treatment can help PCOS patients better manage psychological and mood disorders, leading to improved rates of successful insemination [127,128]. However, there is a lack of studies investigating the potential use of herbal remedies in improving eating disorders in PCOS. Acupuncture has been used to enhance metabolic, reproductive, and mental health in women with PCOS [129]. A recent systematic review found potential benefits for metabolic parameters and stress or depression-related tests in women with PCOS. However, it is not easy to draw definitive conclusions because there are differences among studies in terms of acupuncture techniques, treatment duration, and the coadministration of other drugs. Additionally, it has not been studied whether acupuncture can help reduce food cravings and binge eating in women with PCOS [130].

## 7. Conclusions

**Figure 1:**
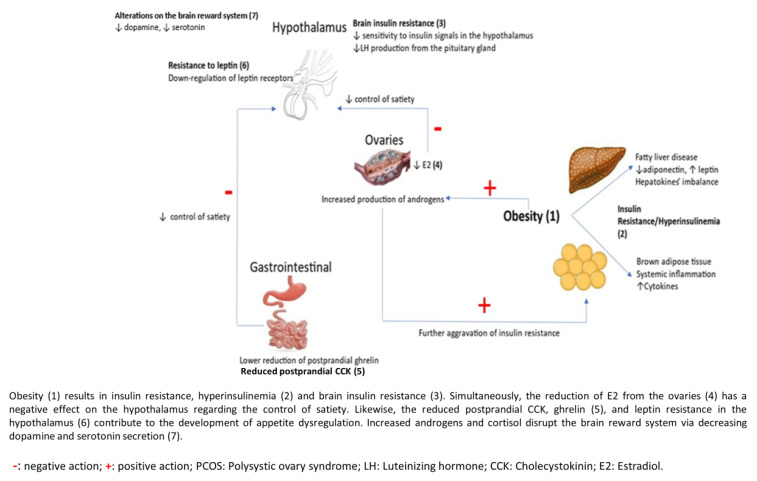
Pathogenetic mechanisms between eating disorders and polycystic ovary syndrome.

PCOS, besides being an endocrine disease, is a metabolic disorder. Hyperinsulinemia and hyperandrogenism contribute to increased caloric intake, food cravings, and fat accumulation. In addition, low self-esteem and impaired body image result in mood disturbances, such as excessive anxiety and depression, which further aggravate food disorders. Normally, hypothalamic signaling regulates appetite and ceases excessive food intake. However, the hypothalamic function is blunted in PCOS and, thus, is incapable of compensating for food surplus (Figure 1). As a result, a vicious cycle of extreme food intake, weight gain, IR, and mood disorders are developed. Though several food and mood disorders have been recognized in women with PCOS, binge eating and depression seem to be the most common. However, it is challenging to identify the prevalence of these disorders, as their diagnosis is usually based on self-questionnaires and not on an evaluation from a specialist. Furthermore, the study population is often heterogeneous in terms of the age of the participants, the diagnostic tests applied, and the duration of follow-up. There are currently no specific recommendations for treating food cravings and binge eating in individuals with PCOS. The efficacy of behavior treatments and medical agents in mitigating overeating in individuals with obesity has been established through various studies. However, there is a lack of research on the impact of these interventions exclusively in women with PCOS and comorbid food disorders. Furthermore, large, well-designed, randomized controlled trials are needed to determine the optimal diet, type, and duration of exercise to alleviate food disorders in women with PCOS.
